# Motor Imagery of Speech: The Involvement of Primary Motor Cortex in Manual and Articulatory Motor Imagery

**DOI:** 10.3389/fnhum.2019.00195

**Published:** 2019-06-11

**Authors:** Gwijde Maegherman, Helen E. Nuttall, Joseph T. Devlin, Patti Adank

**Affiliations:** ^1^Department of Speech, Hearing and Phonetic Sciences, University College London, London, United Kingdom; ^2^Department of Psychology, Lancaster University, Bailrigg, United Kingdom; ^3^Department of Experimental Psychology, University College London, London, United Kingdom

**Keywords:** TMS - motor-evoked potentials, motor imagery (MI), motor execution (ME), time course, primary motor cortex (M1)

## Abstract

Motor imagery refers to the phenomenon of imagining performing an action without action execution. Motor imagery and motor execution are assumed to share a similar underlying neural system that involves primary motor cortex (M1). Previous studies have focused on motor imagery of manual actions, but articulatory motor imagery has not been investigated. In this study, transcranial magnetic stimulation (TMS) was used to elicit motor-evoked potentials (MEPs) from the articulatory muscles [*orbicularis oris* (OO)] as well as from hand muscles [*first dorsal interosseous* (FDI)]. Twenty participants were asked to execute or imagine performing a simple squeezing task involving a pair of tweezers, which was comparable across both effectors. MEPs were elicited at six time points (50, 150, 250, 350, 450, 550 ms post-stimulus) to track the time course of M1 involvement in both lip and hand tasks. The results showed increased MEP amplitudes for action execution compared to rest for both effectors at time points 350, 450 and 550 ms, but we found no evidence of increased cortical activation for motor imagery. The results indicate that motor imagery does not involve M1 for simple tasks for manual or articulatory muscles. The results have implications for models of mental imagery of simple articulatory gestures, in that no evidence is found for somatotopic activation of lip muscles in sub-phonemic contexts during motor imagery of such tasks, suggesting that motor simulation of relatively simple actions does not involve M1.

## Introduction

Motor imagery has been defined as “the mental rehearsal of simple or complex motor acts that is not accompanied by overt movement” (Porro et al., 1996, p.7688). The concept of motor imagery is inherently linked to that of motor execution, and the former is most easily conceptualized as a covert form of—or subset of processes relating to—the latter (Jeannerod, 2001). Fadiga et al. (1998) proposed that motor imagery includes aspects of motor execution experience, on the basis that numerous biological parameters such as blood pressure and heart rate, which increase during action execution, also correlate positively with action effort during motor imagery. Likewise, the time course of motor imagery and motor execution of actions has been found to be similar (Parsons, 1994). Functional imaging had by this time also shown some overlapping activation for both types of processing (Porro et al., 1996; Roth et al., 1996). Since such measures suggest a significant level of shared processing between the two behaviors, the extent to which motor plans are used in motor imagery compared to motor execution has been the subject of debate. Notably, Vogt et al. (2013) proposed a continuum from action observation to motor imagery, along which the extent of the simulation of motor plans differs, though neither effect motor unit activation. Similarly, Jeannerod (2001) proposed that covert and overt action stages also represent a continuum where execution suggests the existence of a covert stage, but covert actions do not evolve into the overt stage. In this sense, it can be said that a continuum of action processing, and so the use of motor plans for potential simulation of action, exists from action observation through covert simulation of motor plans to overt execution of motor plans. Such models often implicate primary motor cortex (M1) as an important element in simulation processing (Tian et al., 2016), but the central question remains at what level, and to what degree, motor imagery makes use of motor plans in the absence of overt action execution.

Early studies into motor imagery made use of mental rotation paradigms and investigated how complexity can affect reaction time. Two well-known examples of this are the Shepard-Metzler visual rotation task (Shepard and Metzler, 1971) and the hand laterality task (Parsons, 1994). In the Shepard-Metzler task, participants are asked whether two three-dimensional shapes (on a two-dimensional plane) are matching objects or not. The task involves the use of mental rotation of this shape so as to try and decide whether two objects correspond or differ. Reaction times tend to be positively correlated with the size of the angle that subjects are required to rotate the object by mentally, so as to be able to provide a yes/no response. Similarly, the hand laterality task presents an image of a left or right hand on-screen, with the participant deciding which hand is shown. Reaction times here tend to be positively correlated with the angle required to move the hand from its canonical position (achieved by lifting the hand in front of the eyes) to that of the hand shown on-screen. Of particular note is that trials involving uncommon angles or difficult flexion in real-world motor execution processes (e.g., right hand rotated laterally rightward by >45°, which requires distinctly more wrist and elbow motion compared to internal leftward rotation of >45°) are modulated to a similar degree in motor imagery, even though no physical constraints exist. These tasks and other similar tasks have been adapted for use with imaging methods such as positron emission tomography (PET; Deiber et al., 1998; Kosslyn et al., 1998) and functional magnetic resonance imaging (fMRI; Porro et al., 1996; Richter et al., 2000; for review, see Munzert et al., 2009). Many of these imaging studies find activation in supplementary motor area (SMA) and premotor areas (Richter et al., 2000; Kosslyn et al., 2001), while some studies also find activation in M1 (Stippich et al., 2002; Solodkin et al., 2004; again see Munzert et al., 2009, p.308 for an extensive overview), and especially early articles (see Porro et al., 1996) disagree on whether motor imagery activates M1.

Several studies have examined the involvement of M1 in motor imagery, primarily in the hand area of M1, using transcranial magnetic stimulation (TMS). For instance, Izumi et al. (1995) asked participants to REST, THINK (motor imagery) or CONTRACT (motor execution) with regard to index finger abduction, with electrodes measuring electrical activity at the *first dorsal interosseous* (FDI—index finger abductor; Izumi et al., 1995). A TMS pulse was administered 3–5 s after the instruction was given. Fifteen motor-evoked potentials (MEPs) were collected per participant per condition. MEPs are responses induced in specific muscles by administering a TMS pulse to the cortical site governing muscle activation on the M1 and reflects the underlying excitability of the neurons at that cortical site and throughout the motor pathway. An increase in MEP amplitude reflects an increased underlying activation of the motor site and pathway and is referred to as facilitation. Significant differences were found between the positively modulated THINK and non-modulated REST conditions, as well as between CONTRACT and THINK conditions and CONTRACT and REST conditions, showing that M1 is active not only during motor execution but also motor imagery. Further studies investigating motor imagery effects find differences between motor imagery and rest conditions in the forearm (Yahagi et al., 1996) as well as biceps and thumb muscles (Fadiga et al., 1998). With respect to other muscles, Tremblay et al. (2001) similarly examined leg muscle responses (quadriceps and thigh muscles) using MEPs and found similar facilitation effects in M1 for imagery respective to non-facilitation during rest. In summary, a number of TMS studies have shown that motor imagery increases the excitability of primary motor cortex as measured by larger MEPs.

While the focus of the above articles is rightly on the increase in MEPs, there is little discussion as to the fine-grained chronometry of motor imagery processes, as few studies included several time points across which MEPs were compared. However, one such study by Hashimoto and Rothwell (1999) made use of TMS to investigate a simple task with flexion and extension of the wrist, measuring MEP amplitude at the *first dorsal interroseous* (FDI; thumb abductor, here used as the control muscle), *flexor carpi radialis* and *extensor carpi radialis* (two muscles engaged in radial abduction of the wrist). Nine participants were told to imagine performing repetitive wrist movements at a rate of 1 Hz, with TMS applied over the hand area of M1 at 0 ms, 250 ms, 500 ms and 750 ms after an auditory cue. Using averages of 8 MEPs, the authors found increases in MEP amplitude over the time course 0–250 ms, with a plateauing and decrease of MEP amplitude at 750 ms in *flexor* and* extensor carpi radialis*. However, the authors did not compare activation in imagery condition with an execution condition. Given the simplicity of the task, it should be possible to replicate such detailed chronometric results for the *first dorsal interosseous* when it is the muscle of interest, as it is here.

Hyde et al. (2017) used the hand laterality task to measure the involvement of M1 in motor imagery of simple and extensive manual rotation using TMS. Hyde et al. (2017) measured MEPs from the hand (FDI) region in M1 to measure its involvement in motor imagery. Single-pulse TMS was administered at latencies of 50 ms, 400 ms and 650 ms post-stimulus presentation. The latter two time points (400 and 650 ms) were included as previous studies (Ganis et al., 2000; Tomasino et al., 2005) showed increased reaction times during the task when TMS was administered at these time points. The early (50 ms) timepoint was added as an early stimulation reference point. Twenty-two participants were tested and subdivided into groups of “likely motor imagery users” and “likely non-motor imagery users” on the basis of whether performance was better in case of simple biomechanical actions (e.g., a 90° internal rotation) than of complex biomechanical action (e.g., a 90° external rotation). Those likely to have used motor imagery were thought to use a strategy whereby they mentally rotated their hand to fit the hand displayed, whereas those likely not to have used motor imagery are thought to have used a strategy whereby they rotated the on-screen picture to fit their hand rather than vice versa. If response times were not modulated on the basis of physical difficulty, it was assumed that a strategy other than motor imagery was used, and subjects were placed in the likely non-motor imagery group. In the likely motor imagery users group, there was an increase in MEP at all three time points (vs. baseline), while this was not the case for likely non-motor imagery users. This increase in MEP during motor imagery presented evidence for increased cortical excitability of hand M1 during imagery of manual movement.

The current study aimed to extend findings of previous work (Hashimoto and Rothwell, 1999; Hyde et al., 2017) on motor imagery in hand muscles by studying the effects of motor imagery for facial muscles, specifically the articulatory muscles, i.e., *orbicularis oris* (OO) in a sub-phonemic context. The sub-phonemic quality of a simple lip task is on par with the non-linguistic nature of a simple hand task—a task containing linguistic elements is eschewed in favor of establishing the viability of basic imagery data collection from both effectors. While motor imagery has been investigated using several hand muscles as well as leg muscles, there is a lack of studies in other muscles—to our knowledge there has been no investigation into articulatory muscles, such as lips or tongue, even though speech imagery is a well-known phenomenon (Sokolov, 1972; McGuire et al., 1996; Oppenheim and Dell, 2008; Alderson-day and Fernyhough, 2015). Moreover, the effect of observation of speech on cortical excitability of lip area is similar to the observation of other motor sequences, e.g., manual sequences (Fadiga et al., 2002; Watkins et al., 2003). Several studies have examined motor imagery of speech actions using fMRI with motor tasks (Huang et al., 2002; Shuster and Lemieux, 2005; Szenkovits et al., 2012; Tian et al., 2016). For instance, Tian and Poeppel (2010, 2012) used fMRI and motor imagery tasks to investigate the topography and time course of imagined speech actions and found that such imagery actions are likely part of a simulation process. This simulation process is likely a sub-process of an internal forward model used in prediction and online correction of movement errors. Manual motor imagery has been investigated with neuroimaging and by measuring MEPs, and motor imagery of speech has been investigated using neuroimaging techniques only. No study so far has thus investigated cortical excitability as measured using MEPs related to motor imagery for speech actions, and as far as we are aware, no study has directly compared cortical excitability of hand and lip actions in a motor imagery context. Sub-cortically, hand and lip MEPs arise *via* different efferent motor pathways: the corticospinal pathway innervates the hand while the corticobulbar pathway innervates the lip muscles (Adank et al., 2018). As a result, there are differences between MEPs recorded from these sites. One difference concerns MEP latency, which is longer in the hand due to the length of the corticospinal tract compared to the corticobulbar tract, resulting in an MEP around 20 ms post-pulse as opposed to 8–10 ms post-pulse in the lip. A second difference concerns MEP morphology, which is single-peaked in the hand but often multi-peaked in the lip. As a result, the MEP extraction method (area-under-the-curve (AUC) vs. peak-to-peak) is critical and should be standardized across studies. Given these differences which have not been addressed in the motor imagery literature, it is necessary to evaluate the effects of motor imagery processes in corticobulbar-innervated muscles such as OO, and whether results from the hand literature can be generalized to such muscles (at least with regard to simple actions).

We examined motor imagery in hand and lip muscles while participants performed a simple motor imagery task. While a number of previous studies used a multi-finger finger tapping task to investigate hand motor imagery, such a task is difficult to extend to the lip muscles due to the smaller number of joints and muscles involved. We opted instead for an imagery task that was simple and could be performed equally with both effectors using simple compression, similar to joint flexion (Hashimoto and Rothwell, 1999). In the study, participants were asked to either imagine pressing together a set of wooden tweezers between their lips (lip motor imagery task), or between their thumb and index finger (hand motor imagery task), or to press together the wooden tweezers between the lips (lip motor execution task), or fingers (hand motor execution task), or to do nothing (remain at constant contraction—baseline). The squeezing together of the lips, in particular, can be considered a sub-phonemic articulatory gesture, used in the production of plosive sounds, such as /p/. In line with Hyde et al. (2017) and Hashimoto and Rothwell (1999), we used a chronometric design and measured MEPs at various time points from the trial onset, including 50, 150, 250, 350, 450 and 550 ms. A wide range of time points was used as it is unclear to which extent motor imagery and motor execution of speech muscles follow the same time course. By examining motor imagery and motor execution in speech and hand muscles we aimed to clarify to which extent primary motor cortex for sub-phonemic movement engages during motor imagery, as well as how this involvement develops over time.

## Materials and Methods

### Participants

Twenty participants (11 F, 9 M, mean age = 22 years 7 months, SD = 3 years 8 months, age range = 19–34) took part in the experiment. Handedness was established *via* the Edinburgh Handedness Inventory (Oldfield, 1971), which found all participants to be right-handed. Participants reported no history of neurological/ psychiatric disease, and none reported the use of any long or short-term medication. No medical conditions, relevant or otherwise, were reported, and neither was any history of specific repetitive motor activity or muscle disorders. All subjects completed all conditions on the same day in one session and had a minimum high school-level education. Experiments were undertaken with the understanding and written consent of each subject, according to the University College London Research Ethics Committee (UREC). We adhered to data collection and reporting conventions as established in Chipchase et al. (2012).

### Materials

**Figure 1:**
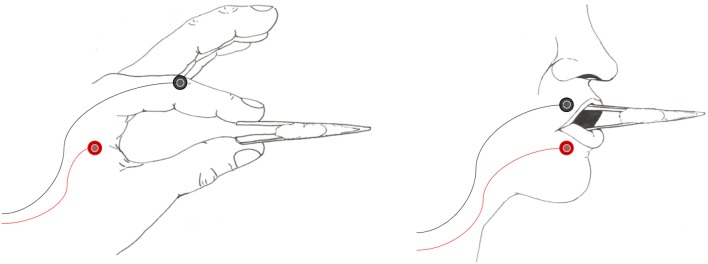
Examples of the tweezers as used in the hand (left panel) and lip (right panel) conditions. Participants were instructed to maintain a baseline level of activity as trained on in a pre-thresholding session. New tools were provided for each participant, with a white cotton filling acting as a spring to provide tension for muscle activation.

We used a tweezer-like tool to effect constant muscle contraction as well as to provide a tool for the motor execution condition (Figure 1). Prompts were displayed on a 21.5” computer monitor screen while participants sat approximately 70 cm away. Prompts were a combination of symbols (font size 24), with “%%” representing hand use and “&&” representing lip use. Colour was used to indicate whether the action should be imagined (blue) or overt (red), or whether no action should be taken (black). Prompts were displayed on a light-gray background, and were preceded by a white fixation cross.

### Procedure

**Figure 2:**
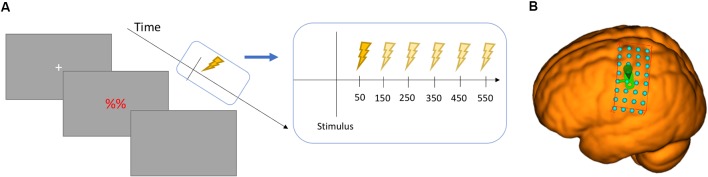
**(A)** Chronometric design of the study showing the six time points in relation to the stimulus. **(B)** the grid surface for the MNI brain used in thresholding.

Each trial began by displaying a white fixation cross for 1,000 ms. The prompt was shown for 2,000 ms, with TMS pulses administered at 50 ms, 150 ms, 250 ms, 350 ms, 450 ms, or 550 ms post-stimulus (counterbalanced). The screen was cleared of all prompts and remained clear for at least 2,000 ms, but longer depending on when the TMS pulse was given. The inter-pulse-interval was kept constant at 6,000 ms. The next trial then began with a new fixation cross (see Figure 2A). Blocks consisted of 25 trials presented in 2 min, with a break of 1 min in between each block. Each break was a minimum of 1 min, after which the second tester pressed a control key to continue unless the participant requested more time for a break. Trials were blocked by effector in separate blocks (first effector counterbalanced across participants), with motor execution, motor imagery and baseline prompts as interleaved, mixed trials to avoid potential muscle activation carry-over effects from one stimulus to the next. Each prompt was presented 15 times so that 15 MEPs could be used to derive an average for each trial type at each of the chronometric time points (see Figure 2A). The experiment consisted of 450 trials in total (150 per task, 30 per time point). The experiment lasted 2 h (45 mins of TMS).

Participants were recruited using the UCL online participant pool by advertising the study as a session which examined the effects of attention on task processing without explicit reference to MEPs, reaction time, grip strength, or imagery.

Upon arrival, the study was explained to the participant, and they were given information about the TMS procedure. The participant was then shown what they were expected to do for each symbol. A training session showed each trial type four times allowing the experimenter to make comments on the actions performed. Training lasted 2 min and all participants were able to successfully perform the actions.

Upon completion of the TMS section of the study, an audiometry test was completed (tested at 0.5, 1, 2, 4, and 8 kHz, in accordance with guidelines from the British Society of Audiology (2011), as was a Montreal Cognitive Inventory test (Nasreddine et al., 2005) to exclude any abnormalities. Finally, participants completed the Varieties of Inner Speech Questionnaire (VISQ, McCarthy-Jones and Fernyhough, 2011). The VISQ was included to test for a potentially positive correlation between any VISQ factors (level of condensed inner speech, level of dialogic inner speech, level of other people represented in inner speech and level of motivational inner speech) and MEP AUC, as it is expressly designed to assess the nature of inner speech and the extent to which it is used on a per-subject level.

All MEPs were initially extracted from Spike^2^ using custom-made scripts that allowed for the extraction of the electromyography (EMG) signal for a time-frame between 1,000 ms pre-TMS pulse and 40 ms post-TMS pulse. The AUC of each MEP was then calculated, with hand MEPs spanning the region 13–40 ms post-TMS pulse, and lip MEPs spanning the region 8–35 ms post-TMS pulse due to their shorter onset time. An equally long section of 27 ms was extracted pre-pulse to allow *post hoc* checks of equivalent baseline contraction across conditions. The first MEP in each block was then removed as the first MEP is non-representatively larger than those that follow, and this ensures stable neuronavigated coil placement is in place. The mean and standard deviations for the data of each effector was calculated and used to standardize the results and so enable cross-effector comparisons (*z*-scores).

### Transcranial Magnetic Stimulation

Following the successful conclusion of the training session, the electrodes (Ag/AgCl, ø10 mm) were attached to the FDI (tendon-belly montage) and the OO (belly-belly montage), and 2 min were used for training to maintain baseline activity at 20% of maximum voluntary contraction. This was intended to ensure consistent muscle activation maintained throughout the experiment, except when responding in the action condition. The EMG signal was sampled at 5,000 Hz, amplified by a factor of 1,000 and band-pass filtered between 100 and 2,000 Hz using a 1902 amplifier, with digital-to-analog conversion using a Micro1401 unit (both Cambridge Electronic Design, Cambridge, UK) connected to a Windows 7 PC. Frameless stereotaxy (Brainsight, Rogue Resolutions, Montreal, QC, Canada) was used to localize the area of stimulation for each effector, making use of the built-in MNI-152 model. A virtual 8 × 4 grid was placed over the motor cortex with the center corresponding to MNI coordinates −64, −4, 39 (see Figure 2B). This was done to search for the hand and lip motor areas in a simple and consistent manner. This system was also used to ensure correct coil location and stability were maintained throughout. A figure-of-eight coil (ø70 mm) was placed at a 45° angle relative to the sagittal plane, inducing a posterior-anterior current flow approximately perpendicular to the lateral fissure. Once all grid points had been given a minimum of three TMS pulses, the MEPs were extracted from Spike^2^, averaged and visualized using a heatmap display command in MATLAB, displaying the best area to stimulate for each effector. Thresholding was performed using a standard thresholding procedure in which 5/10 MEPs must be elicited (Rossini et al., 1994, 2015; Watkins et al., 2003). An MEP was defined as a peak-to-peak amplitude of 500 μV for FDI and 200 μV for OO. Once active motor threshold (aMT) was established, testing threshold was set to 120% of aMT. This resulted in effector-specific motor threshold and testing intensities as necessitated by the inherent differences between the motor thresholds of facial and manual muscles (Groppa et al., 2012).

### Analysis

We first performed a repeated-measures analysis of variance (ANOVA) on the combined lip and hand data to establish the effect of the factors Effector (hand vs. lip), Condition (motor execution, motor imagery, baseline) and Time point (50, 150, 250, 350, 450 and 550 ms) on the AUC of MEPs. This overall ANOVA was followed up by two separate ANOVAs for each effector separately to investigate the key interactions. Follow-up *t*-tests were also performed. All results were Greenhouse-Geisser corrected where assumptions of sphericity were violated. All significance values were corrected for multiple comparisons (Bonferroni) where applicable. MEPs were removed from the analysis if they exceeded 3 SDs from the effector-specific mean, which resulted in the removal of 64 MEPs (~0.7%). No subject scored <26 in the Montreal Cognitive Assessment, indicating normal cognitive function in all participants.

## Results

### Overall Data

Average MNI coordinates for hand were (−40, −15, 67) and average MNI coordinates for lip were (−59, −8, 46; see Supplementary Table S1). Due to issues with the frameless stereotaxy system, we were able to extract localization data from 13 out of 20 participants. A one-way ANOVA comparing baseline muscle contraction levels found no significant differences in baseline contraction across conditions in either the hand (motor imagery mean = 0.22 mV/ms, SD = 0.10 mV/ms; motor execution mean = 0.22 mV/ms, SD = 0.10 mV/ms; baseline mean = 0.21 mV/ms, SD = 0.10 mV/ms; *F*_(2,357)_ = 0.058, *p* = 0.943, n.s.) or lip (motor imagery mean = 0.37 mV/ms, SD = 0.19 mV/ms; motor execution mean = 0.38 mV/ms, SD = 0.19 mV/ms; baseline mean = 0.37 mV/ms, SD = 0.19 mV/ms; *F*_(2,357)_ = 0.095, *p* = 0.910, n.s.), showing that any difference in MEPs was not due to different levels of pre-pulse contraction.

**Figure 3:**
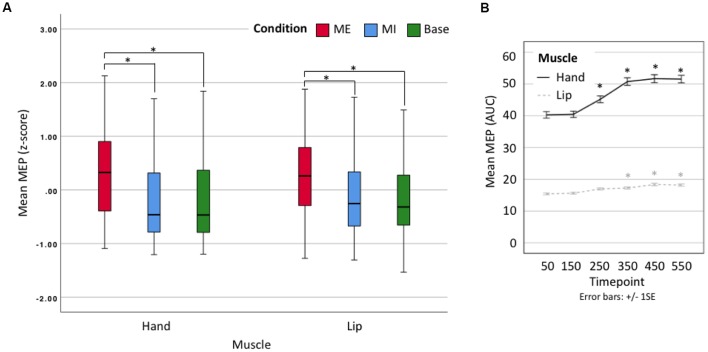
**(A)** Box plot showing *z*-scores for all data. Lip results appear as smaller due to their naturally smaller size. ME, motor execution; MI, motor imagery; Base, baseline. *Denotes significant difference. **(B)** Line plot showing mean motor-evoked potential (MEP) area-under-the-curve (AUC; raw) by timepoint. Error bars represent ± 1 SE, *α* = 0.001. *Denotes significant difference with respect to first time point.

We proceeded to use *z*-scores of the averaged data for a 2 × 3 × 6 omnibus ANOVA comparing Effector (hand vs. lip), Condition (motor execution, motor imagery, baseline) and Time point (50, 150, 250, 350, 450, 550). There was no significant main effect of Effector (*F*_(1,19)_ = 0.014, *p* = 0.906, n.s.) as expected given our use of *z*-scores, but there was a significant main effect of Condition (*F*_(1.1,20.9)_ = 54.202, *p* < 0.001, $\eta_{\text{p}}^{2}$ = 0.740). *Post hoc* comparisons show that motor execution MEPs (Mean = 0.297, Standard Error = 0.097) were significantly larger than motor imagery (*M* = −0.177, SE = 0.122, *p* < 0.001) and baseline MEPs (*M* = −0.188, SE = 0.116, *p* < 0.001), but that there was no difference between motor imagery and baseline MEPs (*p =* 1; see Figure 3A). A second main effect was found for Time point (*F*_(2.6,49.4)_ = 22.273, *p* < 0.001, $\eta_{\text{p}}^{2}$ = 0.540). *Post hoc* comparisons showed a significant difference between the first timepoint and all subsequent time points (minimum Mean Difference = −0.166, SE = 0.036, *p* = 0.003), except timepoint 150 (MD = −0.030, SE = 0.022, *p* = 1; see Figure 3B). There was also a significant Condition × Time point interaction (*F*_(3.8,71.4)_ = 24.651, *p* < 0.001, $\eta_{\text{p}}^{2}$ = 0.565) with no other interactions found. Having shown that there is no difference between the effectors after standardization around the mean (*z*-scores) we used the raw data in our per-effector analyses.

### Lip

**Figure 4:**
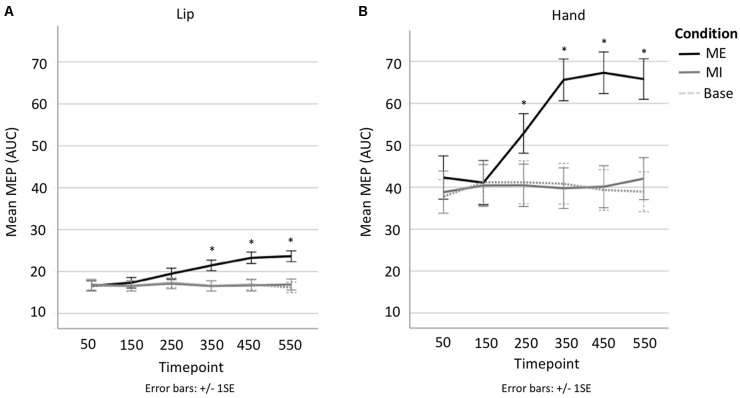
**(A)** Line plot showing change in MEP AUC per time point per condition in lip. **(B)** line plot showing change in MEP AUC per time point per condition in hand. *Denotes significant difference with respect to first time point.

A 3 × 6 repeated-measures ANOVA comparing Condition (motor execution, motor imagery, baseline) and Time point (50, 150, 250, 350, 450, 550 ms) was run on the lip data. The analysis of the lip MEPs showed a main effect of Condition (*F*_(1.2,22.8)_ = 30.999, *p* < 0.001, $\eta_{\text{p}}^{2}$ = 0.620), with contrasts revealing motor execution MEPs to be significantly larger than motor imagery MEPs (MD = 3.512, SE = 0.582, *p* < 0.001) and baseline MEPs (MD = 3.583, SE = 0.647, *p* < 0.001), but with no significant difference found between the motor imagery and baseline conditions (MD = 0.070, SE = 0.233, *p* = 1). There was also a main effect of Time point (*F*_(2.9,54.4)_ = 10.893, *p* < 0.001, $\eta_{\text{p}}^{2}$ = 0.364), with contrasts revealing MEPs at 50 ms to be significantly smaller than at 350 ms (MD = −1.526, SE = 0.418, *p* = 0.025), 450 ms (MD = −2.287, SE = 0.538, *p* = 0.006) and 550 ms (MD = −2.234, SE = 0.478, *p* = 0.002; see Figure 4). This main effect, however, was entirely driven by a significant interaction between Condition × Time point (*F*_(3.9,73.8)_ = 12.630, *p* < 0.001, $\eta_{\text{p}}^{2}$ = 0.399). Specifically, the MEP size only increased with time in the motor execution condition but not in the motor imagery or baseline conditions (see Figure 4B). Planned follow-up tests confirmed that while there were no differences across time points in the motor imagery and baseline conditions, there were significant differences across time points in the motor execution conditions, with MEPs at 50 ms significantly smaller at than at 250 ms (*t*_(19)_ = −3.894, *p* = 0.001), 350 ms (*t*_(19)_ = −4.543, *p* < 0.001), 450 ms (*t*_(19)_ = −5.466, *p* < 0.001), and 550 ms (*t*_(19)_ = −6.591, *p* < 0.001; see Supplementary Table S3). In other words, MEP size only changed when participants actually squeezed the tweezers with their lips, and not when they only imagined doing so.

### Hand

A 3 × 6 repeated-measures ANOVA comparing Condition (motor execution, motor imagery, baseline) and Time point (50, 150, 250, 350, 450, 550 ms) was run on the hand data. There was a main effect of Condition (*F*_(1.1,21.3)_ = 46.083, *p* < 0.001, $\eta_{\text{p}}^{2}$ = 0.708), indicating that motor execution MEPs were significantly larger than motor imagery MEPs (MD = 15.549, SE = 2.285, *p* < 0.001) and Baseline MEPs (MD = 15.944, SE = 2.267, *p* < 0.001), but with no significant difference found between the motor imagery and baseline conditions (MD = 0.396, SE = 0.638, *p* = 1; see Figure 3). There was also a main effect of Time point (*F*_(2.4,46.1)_ = 14.491 *p* < 0.001, $\eta_{\text{p}}^{2}$ = 0.433), with contrasts revealing MEPs at 50 ms to be significantly smaller than at 250 ms (MD = −5.186, SE = 1.116, *p* = 0.003), 350 ms (MD = −9.089, SE = 1.421, *p* < 0.001), 450 ms (MD = −9.282, SE = 1.702, *p* < 0.001) and 550 ms (MD = −9.301, SE = 2.008, *p* = 0.003; see Figure 4). This was once again driven entirely by a significant interaction between Condition × Time point (*F*_(3.5,66.6)_ = 15.162, *p* < 0.001, $\eta_{\text{p}}^{2}$ = 0.444). Hand MEPs only increased with time in the motor execution condition but not in the motor imagery or baseline conditions (see Figure 4A). Follow-up tests confirmed that while there were no differences across time points in the motor imagery and baseline conditions, there were significant differences across time points in the motor execution conditions, with MEPs at timepoint 50 significantly smaller than at 250 ms (*t*_(19)_ = −3.835, *p* = 0.001), 350 ms (*t*_(19)_ = −5.006, *p* < 0.001), 450 ms (*t*_(19)_ = −5.863, *p* < 0.001), and 550 ms (*t*_(19)_ = −5.415, *p* < 0.001; see Supplementary Table S2). In other words, MEP size only changed when participants actually squeezed the tweezers by hand, and not when they imagined doing so, as per the lip conditions.

## General Discussion

This study aimed to examine whether primary motor cortex was facilitated for speech motor imagery by examining a simple motor imagery task in lip and hand muscles. A secondary aim was to describe the time course of both motor imagery and motor execution in speech and hand muscles. We conducted an experiment in which participants performed a speech or manual action, imagined performing the action, or did nothing.

The results showed that primary motor cortex was facilitated during action execution for both effectors, but we could not find evidence supporting the prediction that mental imagery involves M1, therefore not replicating results from earlier studies (Hashimoto and Rothwell, 1999; Hyde et al., 2017). The pattern of increasing MEPs in the results for lip and hand during action execution followed a comparable time course, but differences in AUC of the MEPs were more pronounced for hand muscles.

The effect of action execution in both lip and hand muscles showed that MEPs successfully captured M1 facilitation during execution of actions, with the time course showing the expected increase between 150 and 350 ms, plateauing thereafter. Compared to the lip, it might appear that the hand showed changes in reaction times more quickly given that it was different to baseline at an earlier timepoint, though this is likely to be a result of lip MEP changes being overall smaller rather than a result of actual reaction time differences, as the hand action likely engages a larger pool of motor units relative those engaged in lip.

The results showed no evidence of cortical facilitation for the imagery condition compared to the baseline condition for either effector. This result was unexpected as previous studies focusing on hand actions (Hashimoto and Rothwell, 1999; Hyde et al., 2017) reported cortical facilitation during imagery. Both studies used tasks which are broadly comparable to the task used in the present study, namely a mental rotation task and a mental flexion task. However, it should be noted that Hyde et al. (2017) only reported an increase in MEP amplitudes for imagery compared to baseline for a subset of their participants (namely the ones that were classified as using motor imagery to a higher extent). We attempted a similar approach with our data and split our participants into two groups depending on their VISQ scores using a medial split. However, even for those showing higher VISQ scores, no increase in MEPs was found for the hand data (or the lip data). Also, there was no evidence of a general positive correlation between the VISQ scores and the MEP data across participants for either effector (or condition). The lack of an increase in MEPs related to motor imagery in our results might be attributable to task effects. Specifically, it could be the case that our task did not sufficiently encourage participants to engage in motor imagery. The task was deliberately simplified to allow for a direct comparison across hand and lip muscles. Studies that use a more complex task tend to report larger increases in MEPs, for instance, a study by Roosink and Zijdewind (2010) did find such a modulation of MEPs. In this study, the authors measured the difference in corticospinal excitability between action observation and motor imagery processes to infer the usefulness of these processes in motor rehabilitation. The authors found significant differences between active action observation and motor imagery conditions, with active action observation showing greater MEPs than simple or complex motor imagery conditions (which are not between them significantly different). As a result of their statistical analysis in which the rest condition mean and SD was used to obtain *z*-scores for the experimental conditions, the rest condition was not included in the multi-level analysis, and the authors did not claim that the motor imagery conditions were significantly different to the rest condition. However, the authors did report significant differences between simple and complex actions. Based on the results for a complex task reported in Roosink and Zijdewind, 2010 and the lack of a clear result for simple tasks reported in Hyde et al. (2017), it seems possible that our task was too simple to encourage participants to engage in motor imagery.

With respect to the time course results, it was expected that earlier time points would be associated with smaller MEPs than any later time points. Data from both effectors showed the expected pattern of increasing MEP amplitudes for later time points, which was likely due to motor preparation and motor response. This addressed our secondary aim and clarified the time points post-stimulus at which motor excitability was notably modulated during motor processes. Follow-up analyses showed that this was a result of motor execution and very different from motor imagery, which showed no difference to the baseline at any timepoint.

In sum, TMS can be used to measure changes in cortical excitability associated with overt movement in simple tasks but is perhaps not suitable for measuring motor imagery of movement in simple tasks. There are several possible reasons for the lack of an increase in MEPs associated with motor imagery compared to the baseline condition. The first could be that MEP data can index larger changes in cortical activity for active movement, but not for subtler changes, such as imagery of such movement. While certain studies are able to show increases in blood flow to motor regions during imagery (see Munzert et al., 2009, p. 308), including articulation imagery, direct measurement *via* TMS is more difficult to achieve and is perhaps dependent on effort.

It could also be the case that participants did not engage in motor imagery as requested, which would yield results similar to those above. A number of participants pointed out that while the task was intuitive, imagery was not always attained without difficulty. Another reason could be that while active muscle movement activates corresponding motor regions, imagery of muscle movement does not. In this case, our results would contradict several imaging studies that have found action imagery activates motor regions, but would fit with a number of TMS studies that have found no increase in EMG in motor imagery using simple tasks (e.g., Fadiga et al., 1998; Tremblay et al., 2001; and Yahagi et al., 1996). One final possibility is that the premotor cortex and SMA actively inhibit primary motor cortex in motor imagery, but not motor execution, precisely to ensure that no overt movement occurs. This may, in turn, cancel out the activation of primary motor cortex as it is engaged in motor simulation. While this process would affect MEP size negatively, the fMRI Blood-Oxygen-Level Dependent (BOLD) signal would not dissociate between deactivation and activation, potentially explaining studies that show premotor area activation (e.g., Deiber et al., 1998; Kasess et al., 2008). Additionally, there may be inhibition further downstream (e.g., the brainstem or spinal column) which would not be observable in the MEP.

The design of the study also presented certain limitations. As it is not feasible to consistently obtain lip MEPs without muscle contraction (see Cattaneo and Pavesi, 2014), participants were required to perform active isometric contraction throughout the task. Therefore, as a result, our participants effectively performed two simultaneous tasks, the motor execution and imagery task and the isometric contraction task. As a result, the EMG signal could have masked underlying subthreshold voluntary activity. This issue needs to be addressed in future studies, e.g., by attempting a replication of the hand task without voluntary contraction, so that the consistency of task results between active and relaxed muscles can be further examined. Another possibility is the use of a specialized population with regard to lip muscles, e.g., wood and brass instrumentalists, to explore whether a lower threshold or indeed the use of resting motor threshold is possible. In addition, this specialist population may already routinely engage in motor imagery of lip movements through mental rehearsal, which could be exploited in an imagery task.

This study explored the use of MEPs in measuring the excitability changes in M1 during imagery of simple actions in hand and lip muscles. The use of MEPs to address these questions specifically in lip muscles, using both execution and imagery conditions and recording the time course of excitability changes, represents a combination of research techniques to deliver a novel approach towards investigating motor imagery. Given the limited usability of MEPs (i.e., they can be used to index M1 activation and nerve innervation along the motor pathway, but not other cortical regions), this study does not impact on those models of motor simulation which do not necessarily implicate the M1. Similarly, it should be noted that speech is a complex use of muscle groups, rather than the simple use of one single muscle—further research should be conducted into phonemic-level tasks, both hand- and lip-related, to learn more about how motor imagery may be used in higher-level articulatory performance.

In sum, this study aimed to establish whether motor imagery of simple tasks differed from a baseline and action execution condition at the cortical level for hand and lip muscles, and to assess the time course of cortical changes associated with each effector. The results showed that while action execution of simple tasks could be indexed using TMS and MEPs, the same was not found for motor imagery of these tasks. Similarly, while we found that it was possible to record how excitability increased over the six time points for the action execution condition, a similar increase was not recorded for the motor imagery condition. We conclude that the involvement of motor cortex regions during motor imagery may be dependent on task complexity, and may not be implicated in the simulation of simple tasks. Alternatively, motor cortex involvement may be suppressed prior to the signal arriving at the muscle.

## Ethics Statement

This study was carried out in accordance with the recommendation of the UCL Research Ethics Committee (UREC, #0599.001). All subjects gave written informed consent in accordance with the Declaration of Helsinki and the General Data Protection Act 2018. The protocol was approved by the UCL Research Ethics Committee.

## Author Contributions

GM and PA designed the study. GM collected and analyzed the data and wrote the article. PA, HN and JD assisted in writing the article. HN assisted in amending the article.

## Conflict of Interest Statement

The authors declare that the research was conducted in the absence of any commercial or financial relationships that could be construed as a potential conflict of interest.
