# Supplementary material for: Motor Imagery of Speech: The Involvement of Primary Motor Cortex in Manual and Articulatory Motor Imagery
Source: Front Hum Neurosci. 2019 Jun 11;13:195. doi: 10.3389/fnhum.2019.00195 (PMC6579859; doi:10.3389/fnhum.2019.00195)
Supplement: Supplementary file 1 [file Table_1.pdf]

Supplementary material

Table 1: Individual stimulation sites for hand and lip

Subject	*Hand*			*Lip*		
	x	*y*	*z*	*x*	*y*	*z*
1	-14.578	-26.928	79.99	-59.955	-12.718	48.608
2	-38.448	-24.29	70.99	-53.702	-21.274	61.125
3	-39.44	-19.716	71.284	-65.272	-13.944	41.232
4	-17.921	-13.644	77.395	-46.159	-14.865	64.675
5	-36.854	-23.221	72.234	-58.928	-14.3	51.425
6	-52.538	-15.977	59.691	-52.123	-4.951	51.632
7	-45.306	-18.429	66.456	-61.591	-23.482	52.631
8	-58.561	-13.991	51.963	-64.567	8.534	24.566
9	-42.376	-9.662	66.456	-56.598	-4.003	52.484
10	-45.702	2.7	59.22	-62.142	8	34.555
11	-42.376	-9.062	66.456	-56.556	-6.279	42.758
12	-42.376	-9.065	66.456	-61.057	-2.729	43.593
13	-42.376	-9.062	66.456	-64.382	-1.817	34.194
Overall	-39.912	-14.642	67.311	-58.695	-7.987	46.421

Table 2: Interaction effect follow-up tests for hand – results significant at .003 (adjusted for multiple comparisons)

Source	*Condition*	*Timepoint*	*df*	*t*	*p*
Condition * Timepoint	ME	50ms vs 150ms	19	1.069	.298
		*50ms vs 250ms	19	-3.835	.001
		*50ms vs 350ms	19	-5.006	<.001
		*50ms vs 450ms	19	-5.863	<.001
		*50ms vs 550ms	19	-5.415	<.001
	MI	50ms vs 150ms	19	-1.553	.137
		50ms vs 250ms	19	-.984	.337
		50ms vs 350ms	19	-.940	.359
		50ms vs 450ms	19	-1.273	.218
		50ms vs 550ms	19	-2.358	.029
	DN	50ms vs 150ms	19	-1.484	.154
		50ms vs 250ms	19	-1.652	.115
		50ms vs 350ms	19	-1.231	.233
		50ms vs 450ms	19	-.665	.520
		50ms vs 550ms	19	-.415	.683

Table 3: Interaction effect follow-up tests for lip – results significant at .003 (adjusted for multiple comparisons)

Source	*Condition*	*Timepoint*	*df*	*t*	*p*
Condition * Timepoint		50ms vs 150ms	19	-1.476	.156
		*50ms vs 250ms	19	-3.894	.001
	ME	*50ms vs 350ms	19	-4.543	<.001
		*50ms vs 450ms	19	-5.466	<.001
		*50ms vs 550ms	19	-6.591	<.001
		50ms vs 150ms	19	.611	.548
		50ms vs 250ms	19	-.693	.497
	MI	50ms vs 350ms	19	.975	.342
		50ms vs 450ms	19	.353	.728
		50ms vs 550ms	19	-.155	.878
	DN	50ms vs 150ms	19	.097	.924
		50ms vs 250ms	19	-.726	.477
		50ms vs 350ms	19	.047	.963
		50ms vs 450ms	19	-318	.753
		50ms vs 550ms	19	.575	.572
